# Phenotypic and Genotypic Characterization of West Nile Virus Isolate 2004Hou3

**DOI:** 10.3390/ijms20081936

**Published:** 2019-04-19

**Authors:** Shannon E. Ronca, Rodion Gorchakov, Rebecca Berry, R. Elias Alvarado, Sarah M. Gunter, Kristy O. Murray

**Affiliations:** 1 Department of Pediatrics, National School of Tropical Medicine, Baylor College of Medicine and Texas Children’s Hospital, Houston, TX 77030, USA; rodion.gorchakov@gmail.com (R.G.); rberry@bcm.edu (R.B.); Rojelio.Alvarado@bcm.edu (R.E.A.); sm22@bcm.edu (S.M.G.); kmurray@bcm.edu (K.O.M.)

**Keywords:** West Nile virus, molecular virology, 2004Hou3, animal model, arboviruses

## Abstract

West Nile virus (WNV) is an arbovirus with important public health implications globally. This study characterizes a viral isolate, 2004Hou3, in comparison with the NY99 strain from the original WNV outbreak in New York, USA. NextGen sequencing was used to compare the viral isolates genetically, while wild-type C57/BL6 mice were used to compare pathogenicity and viral persistence. Significant differences in survival and clinical presentations were noted, with minor genetic variations between the two strains potentially offering an explanation. One notable difference is that 5 of 35 mice infected with the 2004Hou3 strain developed hind limb flaccid paralysis, suggesting its possible use as a small animal pathogenesis model for this clinical characteristic often observed in human WN neuroinvasive disease patients but not reported in other animal models of infection. Overall, this study suggests that 2004Hou3 is a less pathogenic strain with potential for use in long-term outcome studies using small animal models.

## 1. Introduction

West Nile virus (WNV) is a mosquito-borne flavivirus that causes serious neurologic disease in many parts of the world. In the United States, recent estimates indicate that approximately seven million individuals have been infected [1]. Although the majority (~80%) of WNV infected patients are asymptomatic, a small proportion (<1%) of symptomatic patients will develop more severe neuroinvasive disease (WNND), characterized by encephalitis, meningitis, and/or acute flaccid paralysis. These patients oftentimes experience long-term complications following infection [2,3,4]. 

In order to gain a more complete understanding of the pathogenesis of WNV, several animal models of WNV have been established using NY385-99 (NY-99), a neurovirulent strain that quickly causes disease and death in animal models [5,6]. This rapid disease onset prevents the study of long-term complications using wild-type mice. Additionally, the circulating WN2002 strain has long replaced the NY-99 strain in the US, and phenotypic differences could make the NY-99 strain less relevant in current research models. Therefore, it is crucial to pursue additional models that allow for the evaluation of contemporary genotypes. 

In 2004, we isolated a unique strain of WNV (2004Hou3) in Houston, TX, which we presumed would be similar to the WN2002 circulating strain. We isolated this novel strain from the spleen of an infected juvenile squirrel presenting with severe paralysis and neurological deficits. The purpose of this study was to compare the genetic composition of the 2004Hou3 strain to NY-99 and evaluating the phenotype in the wild-type mouse model. 

## 2. Results

### 2.1. Sequencing

When comparing 2004Hou3 to NY-99, there are 43 mutations and only five of them are nonsynonymous; all five are in the envelope coding region (Table 1). All amino acid substitutions are within the same class: polar/positive-to-polar/positive (arginine to lysine) amino acids, or similar size hydrophobic-to-hydrophobic amino acids, although one substitution of valine to phenylalanine is observed. 

When the sequence for 2004Hou3 is compared to all other available WNV sequences, including Bird114, using BLAST, there are several nucleotide differences that are unique to 2004Hou3. In the envelope region, the nucleotide alteration at 2428 leading to valine to phenylalanine change is exclusive to 2004Hou3, while those alterations at nucleotides 1244 and 1997 appear separately in other WNV isolates, but never together in the same isolate. Another distinctive feature of the 2004Hou3 sequence is the three single nucleotide polymorphisms (SNPs) in the 3’ untranslated region (UTR) at nucleotides 10,440, 10,496, and 10,703. This alteration at nucleotide 10,440 is totally unique to 2004Hou3, while those at 10,496 and 10,703 appear separately, but never together, in other strains.

### 2.2. Pathogenesis in Mice

**Figure 1:**
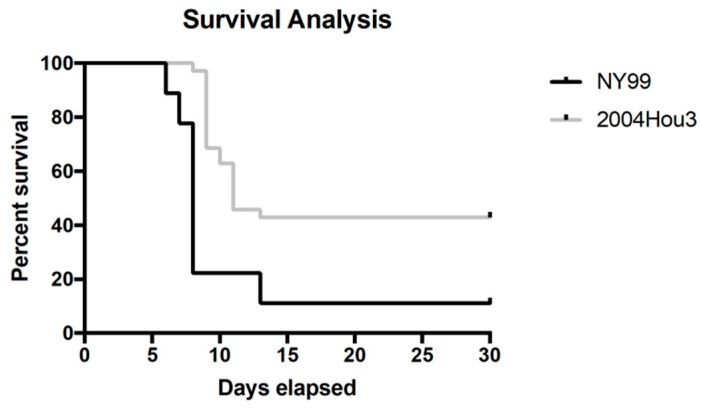
Survival analysis of 2004Hou3 compared to NY-99. Survival was evaluated for a period of 30 days for 2004Hou3 (gray) and NY-99 (black) strains of WNV.

NY-99 infected mice began showing symptoms at 6–7 days post infection (dpi), while WNV 2004Hou3 infected mice had a general symptom onset ranging from 7–13 dpi. WNV 2004Hou3 infected mice displayed hyperactive behavior and acute flaccid paralysis, which were symptoms not observed in NY-99 infected mice. Other observed behaviors were expected. Five 2004Hou3-infected mice (14%) in this study developed hind limb paralysis 5–7 dpi. This paralysis did not greatly affect their ability to move about the cage or retrieve food or water. Of those that developed this paralysis, four reached criteria for euthanasia and one recovered the use of hind limbs and survived through the end of the study. A significant difference was detected in survival using the log-rank test (*p* = 0.0004) and there was an increase in median survival time between the two groups (Figure 1). The median survival time for NY-99 was 8 dpi, compared to the 11 dpi for 2004Hou3. 

### 2.3. Viral Persistance

The persistance of virus in tissues and fluids was evaluated by qPCR. Interestingly, the virus was still detected in the brain, spleen, liver, and kidney 47 dpi and still detected in brain, spleen, and kidney 6 months post infection (mpi) (Table 2). Additionally, the virus was detectable in urine through 47 dpi and detectable in blood as far out of 6 mpi (Table 3).

## 3. Discussion

The study served to characterize the WNV strain 2004Hou3, an isolate from a native Texas squirrel in comparison to the frequently used NY-99 laboratory strain. The sequence of this strain identified only five changes in amino acids in the structural (envelope) region, with both strains’ members of WNV Lineage 1. Although the detected changes are not expected to cause any meaningful changes to protein folding, as most substitutions include amino acids with like characteristics, the most prominent change is the substitution of valine to phenylalanine as a smaller aliphatic to aromatic amino acid substitution. Interestingly, all twelve conserved nucleotide changes of the North American 2002 strain genotype [7] are present in 2004Hou3 and the three substitutions at nucleotides 1465, 6405, and 8235 are the same as some of those found in hamster-passaged strains [8]. These hamster-passaged strains were generated by inoculating hamsters with the NY-99 strain, isolating the virus over time, and using these newly isolated viruses to inoculate additional hamsters to create a viral strain for use in a rodent model [8]. Some of hamster-passaged viruses were previously described to cause persistence in the kidney of mice and mouse cell lines [9,10], which could help to explain the long-term detection of viral RNA in tissue and fluids. 

Although the NY-99 strain is the originally isolated virus from the New York outbreak during the introduction of WNV to the United States, evidence suggests that this strain has been displaced nationwide with those isolated in 2002 [11,12]. Therefore, 2004Hou3 was compared to additional strains, including Bird114 (2002), TX8589 (2012), and BID-V6408/2001 (2001). When compared to other known strains of WNV, 2004Hou3 remains unique. It is most closely related to BID-V6408/2001, which was isolated from the kidney and spleen of an American crow, with 23 SNPs, three of which are nonsynonymous. There are two alterations in 2004Hou3 that are not present in any other WNV strain currently sequenced from any year; one in the envelope region at nucleotide 2428 (V to F) and one in the 3’ UTR at nucleotide 10,440. One could argue that these substitutions could reasonably be enough to be responsible for the altered phenotype observed in mice, or that the synonymous mutations interrupt regulatory RNA regions, but additional research will be necessary. Specifically, viral constructs with individualized substitutions will need to be used in the same mouse model to evaluate outcomes to further delineate the roles of each substitution.

Survival and median time to death were significantly improved in mice infected with 2004Hou3 in comparison to NY-99. This increase of survival using a wild-type mouse model indicates that this strain is a good candidate for long-term small animal studies to evaluate pathways associated with sequelae of infection. Of note, mice infected with 2004Hou3 developed hind limb paralysis only previously described in a hamster model of the NY-99 infection [13,14]. In this hamster model, direct infection of the spinal cord was necessary to observe paralysis. Not only is this route of infection less relevant to the natural infection route, but it may also bypass important innate immune pathways during initial infection [13,14]. Infection with this strain permits the virus to circulate before reaching the brain, replicating a more accurate version of infection, although it does not cause this phenotype in 100% of animals. However, this phenotype variation may be crucial for studying immune and pathological pathways and regulators to determine the pathogenesis of flaccid paralysis. 

Previous studies of chronicity and long-term outcomes in mice have required the use of genetically modified animals [15], or a genetically modified virus [16]. Although this procedure is important for evaluating specific pathways, it may potentially disrupt other important pathways up- or downstream, forcing us to lose valuable information when we knock out the gene of a model animal or the virus. For example, an important study recently identified that complement and microglia are important to memory impairment post WNV-infection, but this required the use of a modified strain of WNV [16]. Use of 2004Hou3 will allow us to study similar outcomes and compare these results to the current literature, providing us with valuable data from a naturally occurring strain of WNV that has not been lab manipulated or attenuated. 

In conclusion, this study describes the initial validation of an alternative mouse model of WNV infection with potential for use in evaluating long term outcomes. Additional studies are necessary to delineate the specific causes of attenuation and evaluate applicable outcome models with this novel strain.

## 4. Materials and Methods

### 4.1. Cells and Viruses

Vero cells (ATCC CCL-81, Manassas, VA, USA) were used for all cell culture experiments. Cells were maintained in complete media. NY385-99 (NY-99) was obtained from R. Tesh (UTMB reference collection, Galveston, TX, USA) and 2004Hou3 was isolated from the spleen of a squirrel collected in Houston, TX (GenBank KC928260.1).

### 4.2. Sequencing

All sequencing was completed at the Alkek Center for Metagenomics and Microbiome Research (Baylor College of Medicine, Houston, TX, USA). RNA was sheared and reverse transcribed using random hexamer primers. Following adapter ligation, cDNA was sequenced on the Illumina MiSeq platform (Illumina, Inc., San Diego, CA, USA) using the 2 × 250 paired-end protocol. Resulting sequencing reads were trimmed to remove adapters and low quality regions. Read pairs in which one or both trimmed reads were less than 50 bp were removed, yielding a final 12,560,165 quality read pairs. Pairs were then screened to further remove PhiX Illumina control (0.001%) and primate contamination (92.15%). To effectively remove the primate signal, genomes of both *Chlorocebus sabaeus* and *Macaca mulatta* were employed. To further depress any remaining primate signal, a subset of 70,000 read pairs was used for genome assembly. The most significant scaffold from a de novo assembly using SPAdes v3.10.1 produced a 10,955 bp contig that aligned to WNV via NCBI BLASTn.

### 4.3. Animals

Female wild-type (WT) C56BL/6 mice aged 4–5 weeks were ordered from Taconic Inc., (B6-F, MPF). Transponders (BMDS IPTT-300) were inserted subdermally for identification and monitored using the BMDS DAS-7007R. Data were collected through the DAS-6001 data acquisition system. Mice were infected with 1 × 10^5^ pfu of 2004Hou3 (N = 35) or NY-99 (N = 9) or mock infected with PBS (N = 6) at 6–7 weeks of age. For infection, virus was diluted in sterile PBS.

### 4.4. Viral RNA Isolation and PCR of Animal Tissue

Tissue was frozen at −80 °C until processing. Tissue was homogenized in complete media and inactivated 1:1 in 2X DNA/RNA shield (Zymo Research, Irvine, CA, USA) and RNA extraction was performed using the Quick-DNA/RNA Viral Kit (Zymo Research). 5 µL of extracted RNA from each sample was used in a single 20 µL rRT-PCR reaction with TaqMan Fast Virus 1-Step Master Mix (Thermo Fisher Scientific, Waltham, MA, USA) on a ViiA 7 Real Time PCR System (Thermo Fisher Scientific) with a TaqMan WNV NS1 assay developed in our laboratory for WNV detection. A separate RT-qPCR reaction was run in parallel for each sample with a generic β-actin assay [17] as the internal positive control for extraction and absence of PCR inhibition. 

### 4.5. Statistical Analyses

Survival analysis was generated with the Kaplan-Meier method using log-rank to compare survival rates. Viral organ loads were determined and compared for each organ with a student’s t test. All graphs were generated using GraphPad Prism version 8 and all analyses performed using IBM SPSS Statistics 25.

## Figures and Tables

**Table 1:** Variations of NY-99 to 2004Hou3. ^1^

Nucleotide position	NY-99 nucleotide (amino acid)	2004Hou3 nucleotide (amino acid)	Genome region
660	C (I)	U (I)	prM
774	U (T)	C (T)	prM
1244	G (R)	A (K)	E
1442	U (V)	C (A)	E
1465	C (L)	U (F)	E
1797	C (N)	U (N)	E
1815	G (S)	A (S)	E
1997	C (A)	U (V)	E
2049	U (F)	C (F)	E
2253	A (G)	G (G)	E
2428	G (V)	U (F)	E
2466	C (H)	U (H)	E
4107	C (A)	U (A)	NS2A
4146	A (L)	G (L)	NS2A
4803	C (R)	U (R)	NS3
5275	C (L)	U (L)	NS3
5280	G (A)	A (A)	NS3
5736	C (C)	U (C)	NS3
6045	C (H)	U (H)	NS3
6138	C (F)	U (F)	NS3
6238	C (L)	U (L)	NS3
6405	U (D)	C (D)	NS3
6426	C (H)	U (H)	NS3
6589	A (R)	C (R)	NS4A
6726	C (V)	U (V)	NS4A
6996	C (F)	U (F)	NS4B
7416	C (P)	U (P)	NS4B
7938	U (G)	C (G)	NS5
7977	A (E)	G (E)	NS5
8199	C (H)	U (H)	NS5
8235	U (C)	C (C)	NS5
8325	C (S)	U (S)	NS5
8520	G (K)	A (K)	NS5
8727	U (T)	C (T)	NS5
8835	G (A)	A (A)	NS5
8970	U (D)	C (D)	NS5
9013	C (L)	U (L)	NS5
9144	C (F)	U (F)	NS5
9352	C (L)	U (L)	NS5
10,338	C (D)	U (D)	NS5
10,440	A	G	3’UTR
10,496	T	C	3’UTR
10,703	G	A	3’UTR

^1^Table 1 Footnote: prM: precursor membrane protein, E: envelope protein, NS2A: nonstructural protein A, NS3: nonstructural protein 3 containing serine protease, RNA helicase, and NTPase, NS4A: nonstructural protein 4A, NS4B: nonstructural protein 4B, NS5: nonstructural protein 5 containing RNA-dependent RNA polymerase, and 3’UTR: 3’ untranslated region.

**Table 2:** 2004Hou3 Viral Persistence in Organs. ^1^

Days Post Infection	Brain	Lung	Heart	Spleen	Liver	Kidney	Bladder
47	4/6	0/6	0/6	2/6	1/6	2/6	NA
180	1/8	0/3	0/3	1/8	0/8	1/8	0/8

^1^Table 2 Footnote: Numbers are presented as total positive over total tested for each time point and tissue type. NA: not available.

**Table 3:** 2004Hou3 Viral Persistence in Mouse Fluids ^1.^

Days Post Infection	Whole Blood	Urine
47	5/6	1/6
60	3/6	0/6
90	3/6	0/6
180	2/6	0/6

^1^Table 3 Footnote: Numbers are presented as total positive over total tested for each time point and fluid.

## Abbreviations

DPI	Days post infection
MPI	Months post infection
PFU	Plaque forming units
SNP	Single nucleotide polymorhpisms
UTR	Untranslated region
WNV	West Nile virus
WNF	West Nile fever
WNND	West Nile neuroinvasive disease
